# Vitamin D level in relation to depression symptoms during adolescence

**DOI:** 10.1186/s13034-022-00489-4

**Published:** 2022-06-27

**Authors:** Reem Al-Sabah, Abdullah Al-Taiar, Lemia Shaban, Ahmed N. Albatineh, Reem Sharaf Alddin, Praveen K. Durgampudi

**Affiliations:** 1 Department of Community Medicine and Behavioral Sciences, Faculty of Medicine, Kuwait University, Kuwait, Kuwait; 2 School of Community & Environmental Health, College of Health Sciences, Old Dominion University, 4608 Hampton Blvd, 3136 Health Sciences Building, Norfolk, VA 23508 USA; 3 Department of Food Science and Nutrition, College of Life Sciences, Kuwait University, Kuwait, Kuwait; 4 CONRAD, Department of Obstetrics and Gynecology, Eastern Virginia Medical School, Norfolk, United States

**Keywords:** Depression, Vitamin D, Adolescents, Kuwait

## Abstract

**Background:**

This study aimed to investigate the association between 25-hydroxyvitamin D (25(OH)D) and depression symptoms among adolescents in Kuwait, a country with a high prevalence of vitamin D deficiency.

**Methods:**

A school based cross-sectional study was conducted on randomly selected 704 adolescents in middle schools. Data on depression symptoms were collected using the Children’s Depression Inventory (CDI). Data on covariates were collected from the parents by self-administered questionnaire and from adolescents by face-to-face interview. Blood samples were analyzed in an accredited laboratory; and 25(OH)D was measured using liquid chromatography-tandem mass spectrometry.

**Results:**

Of 704 adolescents, 94 (13.35%; 95%CI:10.35–17.06%) had depression symptom (a score of 19 or more on the CDI). There was no significant difference in the median CDI score between different vitamin D status (p = 0.366). There was also no significant correlation between serum 25(OH)D concentration and CDI score (Spearman’s rank correlation = 0.01; p = 0.825).There was no significant association between 25(OH)D and depression symptoms whether 25(OH)D was fitted as a continuous variable (crude odds ratio (OR) 0 .99 [95%CI: 0.98, 1.01], p = 0.458 and adjusted OR 1.01 [95%CI: 0.99, 1.02], p = 0.233), categorical variable as per acceptable cut-of points (crude analysis p = 0.376 and adjusted analysis p = 0.736), or categorical variable as quartiles (crude analysis p = 0.760 and adjusted analysis p = 0.549).

**Conclusion:**

Vitamin D status does not seem to be associated with depression symptoms among adolescents in our setting. Nevertheless, it is important to have sufficient vitamin D levels during adolescence for several other health benefits.

## Introduction

The global burden of disease study demonstrated that depression is a major public health problem and is the leading cause of disability worldwide [1]. Depression affects more than 280 million individuals causing great economic burden not only on the affected individuals but also, on their families, communities, employers, and healthcare services [2]. Furthermore, interventions that aim to prevent or improve the outcome of depression have limited success [3] highlighting the need to expand the knowledge on potential risk factors which might influence the risk and the pathogenesis of depression symptoms. The literature suggests that life style factors such as diet [4, 5] and physical activity are related to depression symptoms [6, 7]. Recently, the link between the deficiency in specific nutritional elements such as vitamin D and depression has come under intense debate.

Vitamin D has a well-established role in calcium and phosphorus homeostasis, bone health and various cellular and neuromuscular functions [8]. But recent literature linked low vitamin D levels to several adverse health outcome such as cancer [9], type 2 diabetes [10], multiple sclerosis [11], autism [12], and asthma [13] as well as all-causes mortality [14]. Recently, low vitamin D levels have been linked to the risk [15] and severity [16] of COVID-19. Vitamin D is derived mainly through exposure to sunlight and the link between depression and the lack of sunlight exposure was first noted two thousand years ago [17]. There are several plausible mechanisms in which vitamin D may affect mental health. Vitamin D can cross the blood–brain barrier [18], hence activate the vitamin D receptors (VDR) which are expressed in brain cells along with vitamin D metabolizing enzymes [19–21]. Also, vitamin D is involved in signaling cascades and neurobiological pathways [20] which may affect mental health. The active metabolite of vitamin D is thought to modulate the differentiation and maturation of dopaminergic neurons [22] and to affect brain serotonin concentrations [20, 23]. Finally, it is postulated that depression is associated with increased inflammatory markers [24], while vitamin D has been shown to down-regulate inflammatory markers that have been linked to stress and depression [25]. Despite the evidence that vitamin D may play an important function in the human brain, the exact biological mechanisms linking vitamin D to depression are still not fully understood and remain controversial.

Several observational studies have suggested an association between vitamin D deficiency and major depressive disorders in adults (reviewed by Anglin et al. [26] and Ju et al. [27]). The observational nature of these studies means that it is difficult to ascertain the temporal relationship between vitamin D and depression as some individuals with depression may avoid outdoor activities and have poor diet, resulting in reduced sunlight exposure and consequently reduced endogenous vitamin D synthesis, as well as reduced dietary vitamin D intake [28, 29]. Trials on vitamin D supplementation and depression in adults showed controversial results (reviewed by Cheng et al. [30], Gowda et al. [31], Li et al. [32], Spedding et al. [33], Shaffer et al. [34], and Vellekkatt et al. [35]). In children and adolescents, a recent literature review of observational and interventional studies concluded that there might be a positive influence of vitamin D on mental health in children [36]. The reviewed studies were controversial and heterogeneous which preclude making a robust conclusion and the authors highlighted the need for more studies to facilitate comparisons and deepen the observations.

We have previously demonstrated that 81% of schoolchildren in Kuwait were vitamin D deficient [37]. In this study, we aimed to estimate the prevalence of depression symptoms and investigate the association between Serum 25-hydroxyvitamin D (25(OH)D) and depression symptoms among schoolchildren in Kuwait, a country with plenty of sunshine.

## Methods

### Participants

The study was conducted within a project that aimed to assess vitamin D status in middle schoolchildren (11–16 years) in Kuwait. The details of the project have been described previously [37, 38]. In this project, a nationally representative sample of middle schoolchildren was selected using probability proportional to size sampling method. Trained nurses drew blood samples while trained data collectors gathered data on factors related to lifestyle such as physical activity, sleeping habits, and smoking through face-to-face interviews. Data on socio-demographic factors were collected from the parents of sampled schoolchildren through a self-administered questionnaire.

Another team of researchers at a later time was formulated to interview the schoolchildren and collect data on depression symptoms and its related factors. This was conducted on a subgroup of students (N = 704). Parents provided written informed consent while schoolchildren provided verbal assents before data collection. Both the Ethics Committee at the Health Sciences Centre, Kuwait University (No: DR/EC/2338) and the Ethics Committee at The Ministry of Health in Kuwait (No: 2015/248) approved the study.

### Collection of blood samples and laboratory methods

Serum 25-hydroxyvitamin D (25(OH)D) level was measured using liquid chromatography-tandem mass spectrometry (LC–MS/MS), which is the gold standard method to assess vitamin D status [39, 40]. Other biological measurements including complete blood count, Parathyroid Hormone (PTH), vitamin B12, Iron, ferritin, transferrin and transferrin saturation were all measured in an accredited clinical biochemistry laboratory in a teaching hospital under strict quality control. Details of these measurements have been described before [37, 38].

### Data collection on depression symptoms

Data on depression symptoms were collected using the Children’s Depression Inventory (CDI) [41], which has been previously translated and adopted in our setting [42]. The CDI is a 27-item self-rated symptom scale instrument, which was originally developed based on Beck’s Depression Inventory (BDI) [43], to screen for depression symptoms in children and adolescents aged 7–17 years. The CDI scores symptoms of depression on five subscales that include negative mood, interpersonal problems, ineffectiveness, anhedonia and negative self-esteem with 27 items scored on a 3-point scale (0: absence of the symptom, 1: moderate symptom, 2: severe symptom). The total CDI score is calculated by summing all items, which ranges between 0 (no depression symptom) and 54 (all depression symptoms exist). Several cut-off scores have been recommended such as “ > 13”, “ > 16” and “ ≥ 19” to indicate elevated depression symptoms in adolescents [44]. It has been suggested that a score of ≥ 19 is an appropriate cut-off point for population-based assessment, with 95% and 96% sensitivity and specificity respectively [45, 46]. Also, several studies have adopted this cut-off score to investigate depression symptoms in community-based studies [47–50].

### Data collection on covariates

Data were collected on socio-economic and lifestyle-related factors using self-administered questionnaire that were sent to the parents. Data on behavioral factors such as physical activity, sedentary lifestyle, sleeping hours during weekdays and weekends were collected by face-to-face interviews with schoolchildren by trained team of data collectors. Data on physical activity were collected by a series of questions that were validated among high school students and showed strong correlation with data objectively measured by accelerometers. The total time spent on physical activity per week was calculated. Similarly, data on sedentary lifestyle were collected using a group of questions about the time spent on watching TV, playing video games, using the internet/computer and setting to read/do homework during weekdays and weekends. Standing height and body weight of the schoolchildren were measured using digital weight and height scale (Detecto®) in a standardized manner by trained data collectors.

### Statistical analysis

Using Epidata Entry software, data were double entered into specifically designed database. Then data were transferred to STATA (StataCorp. 2011. Release 14) for data analysis. We used Spearman’s rank correlation to investigate the linear association between CDI score and 25(OH)D level. Vitamin D status was defined according to the Endocrine Society [51] and the Society for Adolescent Health and Medicine [52] as deficiency < 50 nmol/L; insufficiency 50–75 nmol/L; sufficiency ≥ 75 nmol/L. Kruskal–Wallis test was used to investigate the difference in CDI score across different vitamin D status (severe deficiency, deficiency, insufficiency, sufficiency).

The association between 25(OH)D and depression symptoms (CDI score ≥ 19) was assessed using unconditional logistic regression with adjustment for potential confounders. Separate analyses were performed with 25(OH)D fitted as a continuous variable and as a categorical variable. We categorized 25(OH)D using acceptable cutoff points [51] or quartiles. First, crude odds ratios were calculated, then statistically significant variables at 20% level of significant were introduced sequentially to the model while noting the impact of this on the association between 25(OH)D and depression symptoms. There was no difference in conclusion between this analysis and the analysis that was guided by the theory based on the studies that have investigated factors associated with depression symptoms among adolescents and children [53, 54]. As a sensitivity analysis, we used stepwise logistic regression to explore if the conclusion on the association between 25(OH)D and depression symptoms would be different with stepwise variables selection. Furthermore, we used simultaneous quantile regression to assess the association between 25(OH)D (as a continuous and categorical variable) and CDI score as a quantitative outcome using bootstrap to estimate the standard error with 500 replications. Throughout the analysis, all tests were two tailed and factors that showed p < 0.05 were deemed to be statistically significant.

## Results

**Fig. 1:**
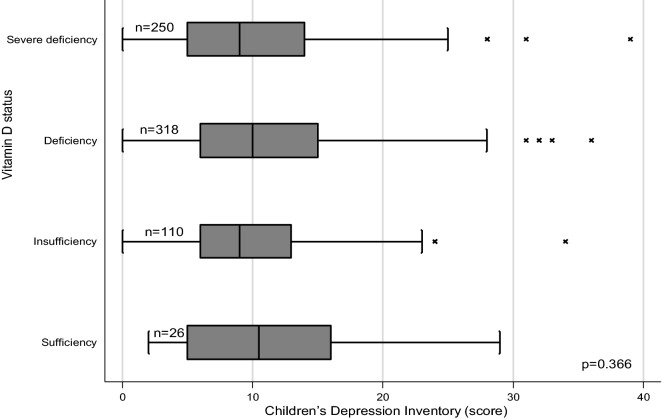
Distribution of Children’s Depression Inventory score by vitamin D status among 704 schoolchildren

The analysis included 704 schoolchildren of whom 353 (50.14%) were males. The mean (standard deviation: SD) age was 12.25 (0.80) years. Of 704 schoolchildren, 94 (13.35%; 95%CI:10.35–17.06%) had depression symptoms (a score of 19 or more on the CDI). Figure 1 shows the distribution of CDI score in different vitamin D status. There was no significant difference in the median CDI score across different vitamin D status (p = 0.366) or different serum 25(OH)D quartiles (p = 0.540). There was also no significant correlation between serum 25(OH)D concentration and CDI score (Spearman’s rank correlation = 0.01; p = 0.825) or between PTH and CDI score (Spearman’s rank correlation = 0.06; p = 0.145).

Table 1 shows the association between socio-demographic factors and depression symptoms (CDI score ≥ 19) in univariable analysis. Only maternal education showed inverse significant association with depression symptoms (p = 0.021). None of the lifestyle factors including the time spent on physical activity (p = 0.461), time spent on sedentary activities (p = 0.714) or body mass index (BMI) categories (p = 0.185) was significantly associated with depression symptoms in univariable analysis (Table 2). However, hours of sleep during weekdays (p = 0.063) and weekends (p = 0.079), and the number of times walking to/from school (p = 0.066) were borderline significantly associated with depression symptoms. Except for PTH, calcium, vitamin B12, anemia, iron, ferritin and folate were all not significantly associated with depression symptoms (Table 3). It is worth noting that the association between PTH and depression symptoms was significant when it was fitted as a continuous variable, but no association was found when PTH was categorized into quartile, tertiles or by the normal value range provided by our laboratory.

**Table 1:** Association between depression symptoms (CDI ≥ 19 score) and socio-demographic factors in adolescents using univariable analysis (N = 704)

Characteristics	Total	Prevalence of depression symptoms		Odds ratio [95% CI]		p
		n*	(%)			
*Gender*						
Male	353	39	(11.05)		[Ref.]	0.071
Female	351	55	(15.67)	1.50	[0.96–2.32]	
*Age (year)*						
< 12	300	46	(15.33)		[Ref.]	0.411
12-	276	33	(11.96)	0.75	[0.46–1.21]	
≥ 13	128	15	(11.72)	0.73	[0.39–1.37]	
*Nationality*						
Kuwaiti	522	73	(13.98)		[Ref.]	0.403
Non-Kuwait	182	21	(11.54)	0.80	[0.48–1.35]	
*School’s Governorate*						
Capital	117	16	(13.68)		[Ref.]	0.962
Hawally	135	16	(11.85)	0.85	[0.40–1.78]	
Farawanya	85	12	(14.12)	1.04	[0.46–2.32]	
Jahra	69	8	(11.59)	0.83	[0 .33–2.05]	
Mubarak al-Kabeer	81	13	(16.05)	1.21	[0.54–2.66]	
Ahmadi	216	29	(13.43)	0.98	[0.51–1.89]	
*Father’s Education*						
Primary/Intermediate/no formal education	95	19	(20.00)		[Ref.]	0.219
Secondary (high school)	167	21	(12.57)	0.57	[0.29–1.14]	
Diploma	145	19	(13.10)	0.60	[0.30–1.21]	
University and above	283	33	(11.66)	0.53	[0.28–0.98]	
*Mother’s Education*						
Primary/Intermediate/no formal education	68	12	(17.65)		[Ref.]	0.021
Secondary (high school)	147	26	(17.69)	1.00	[0.47–2.13]	
Diploma	157	25	(15.92)	0.88	[0.41–1.88]	
University and above	323	29	(8.98)	0.46	[0.22–0.96]	
*Father’s Income (Kuwaiti dinars) *						
< 500	49	3	(6.12)		[Ref.]	0.320
500 to 1000	161	24	(14.91)	2.69	[0.77–9.34]	
1001 to 1500	208	34	(16.35)	3.00	[0.88–10.19]	
1501 to 2000	105	10	(9.52)	1.61	[0.42–6.15]	
More than 2000	95	11	(11.58)	2.01	[0.53–7.56]	
Do not wish to tell	65	8	(12.31)	2.15	[0.54–8.58]	
*Mother employment*						
Housewife	220	32	(14.55)		[Ref.]	0.842
Paid employment	362	47	(12.98)	0.88	[0 .54–1.42]	
Other	110	14	(12.73)	0.86	[0.44–1.68]	
*Living* arrangements						
Lives with the father and the mother	622	79	(12.70)		[Ref.]	0.238
Lives with the mother without the father	60	10	(16.67)	1.37	[0.67–2.82]	
Lives with the father without the mother	19	4	(21.05)	1.83	[0.59–5.66]	
Lives with other relatives	3	1	(33.33)	3.44	[0.31–38.34]	
*Type of housing*						
Rented apartment	275	41	(14.91)		[Ref.]	0.233
Rented house	74	6	(8.11)	0.50	[0.20–1.24]	
Owned apartment	39	8	(20.51)	1.47	[0.63–3.43]	
Owned house	306	38	(12.42)	0.81	[0.50–1.30]	
*Total number of siblings*						
≤ two	177	25	(14.12)		[Ref.]	0.173
Three-four	274	42	(15.33)	1.10	[0.64–1.88]	
Five or more	242	24	(9.92)	0.67	[0.37–1.22]	
*Childbirth order *						
First	192	21	(10.94)		[Ref.]	0.122
Second	147	27	(18.37)	1.83	[0.99–3.39]	
Third or above	351	45	(12.82)	1.20	[0.69–2.08]	
*Passive smoking at home*						
No	462	58	(12.55)		[Ref.]	0.378
Yes	241	36	(14.94)	1.22	[0.78–1.92]	

p-value was generated by Chi-square test of independence or Fisher’s Exact. * Some values are missing, and the total may not add up to 704 for some covariates

**Table 2:** Association between depression symptoms (CDI ≥ 19 score) and lifestyle factors, physical activity, body Mass Index using univariable analysis (N = 704)

Characteristics	Total	Prevalence of depression symptoms		Odds Ratio [95% CI]		p
		n*	(%)			
*Times per week consumed breakfast not prepared at home*						
Zero	320	42	(13.13)		[Ref.]	0.500
One-two times	284	37	(13.03)	0.99	[0.62–1.59)	
Three-four	51	5	(9.8)	0.72	[0.27–1.91]	
Five or more	33	7	(21.21)	1.78	[0.73–4.36]	
*Times per week consumed lunch not prepared at home *						
Zero	185	23	(12.43)		[Ref.]	0.965
One-two times	409	56	(13.69)	1.12	[0.66–1.88]	
Three-four	59	7	(11.86)	0.95	[0.38–2.33]	
Five or more	30	4	(13.33)	1.08	[0.35–3.39]	
*Times per week consumed dinner not prepared at home *						
Zero	81	9	(11.11)		[Ref.]	0.810
One-two times	436	57	(13.07)	1.20	[0.57–2.54]	
Three-four	128	15	(11.72)	1.06	[0.44–2.55]	
Five or more	35	6	(17.14)	1.65	[0.54–5.07]	
*Times per week child has breakfast before going to school*						
Every day/Five days a week	290	42	(14.48)		[Ref.]	0.457
Three-four days a week	94	16	(17.02)	1.21	[0.64–2.27]	
One-two days a week	118	13	(11.02)	0 .73	[0.38–1.42]	
Never	193	22	(11.40)	0.76	[0.44–1.32]	
*Hours of sleep during weekdays*						
< 7.5 h (lower tertile)	205	37	(18.05)		[Ref.]	0.063
7.5 h to < 9 h (middle tertile)	251	29	(11.55)	0.59	[0.35 -1.00]	
9 h or more (higher tertile)	248	28	(11.29)	0.58	[0.34–0.98]	
*Hours of sleep during weekend*						
< 9 h (lower tertile)	149	24	(16.11)		[Ref.]	0.079
9 h to < 11 h (middle tertile)	315	32	(10.16)	0.59	[0.33 -1.04]	
11 or more (middle tertile)	239	38	(15.90)	0.98	[0.56–1.72]	
*Walking to school per week (going and coming equal 2 times)*						
None	562	80	(14.23)		[Ref.]	0.066
1 to 8 times	97	6	(6.19)	0.40	[0.17 -0.94]	
Every day	45	8	(17.78)	1.30	[0.58–2.90]	
*Time spent on physical activity per week*						
Low (lower tertile)	224	33	(14.73)		[Ref.]	0.461
Medium (middle tertile)	234	26	(11.11)	0.72	[0.42–1.25]	
High (higher tertile)	246	35	(14.23)	0.96	[0.57–1.60]	
*Time spent on watching TV, using internet/computer, plying video games and reading or doing homework*						
Low (lower tertile)	224	27	(12.05)		[Ref.]	0.714
Medium (middle tertile)	241	32	(13.28)	1.12	[0.64–1.93]	
High (higher tertile)	239	35	(14.64)	1.25	[0.73–2.14]	
*Body Mass Index categories*						
Normal weight	303	40	(13.20)		[Ref.]	0.185
Overweight	154	14	(9.09)	0.66	[0.34–1.25]	
Obese	235	39	(16.60)	1.31	[0.81–2.11]	
Under weight	12	1	(8.33)	0.60	[0.08–4.76]	

CDI: Children’s Depression Inventory. p-value was generated by Chi-square test of independence. * Some values are missing, and the total may not add up to 704 for some covariates

**Table 3:** Association between depression symptoms (CDI ≥ 19 score) and parathyroid hormone, calcium, vitamin B_12_, anemia, ferritin and folate using univariable analysis (N = 704)

Characteristics	Total	Prevalence of depression symptoms		Odds Ratio [95% CI]		p
		n	(%)			
*Parathyroid Hormone (nmol/L)*	703	–	–	1.04	(1.01- 1.09)	0.023
*Calcium*						
≥ 2.1 (mmol/L)	686	90	(13.12)		[Ref.]	0.213
< 2.1 (mmol/L)	17	4	(23.53)	2.04	[0.65–6.39]	
*Vitamin B12*						
≥ 148 (pmol/L) sufficient	589	76	(12.90)		[Ref.]	0.954
< 148 (pmol/L) deficient	24	3	(12.5)	0.96	[0.28–3.31]	
*Anemia as defined by WHO *[82]						
No	654	85	(13.00)		[Ref.]	0.507
Yes	49	8	(16.33)	1.31	[0.59- 2.88]	
*Iron*	703	–	–	0.98	[0.94–1.02]	0.301
*Ferritin*						
Normal ≥ 15 ng per mL	508	69	(13.58)		[Ref.]	0.790
Low < 15 ng per mL	195	25	(12.82)	0.93	[0.57—1.53]	
*Folate*	704	–	–	1.00	[1.00–1.00]	0.128

WHO: World Health Organization. p-value was generated by Chi-square test of independence, Fisher’s exact test or Wald test as appropriate. * Some values are missing, and the total may not add up to 704 for some covariates

Table 4 shows the association between 25(OH)D and depression symptoms before and after adjusting for potential confounders. There was no significant association between 25(OH)D and depression symptoms whether 25(OH)D was fitted as a continuous variable (crude analysis p = 0.458 and adjusted analysis p = 0.233), categorized by acceptable cut-of points (crude analysis p = 0.376 and adjusted analysis p = 0.736), or when categorized as quartiles (crude analysis p = 0.760 and adjusted analysis p = 0.549). To confirm these findings, forward and backward stepwise logistic regression were used, and 25(OH)D was not selected in any model. Similarly, quantile regression showed no association between 25(OH)D and CDI score in any model (data are not shown). Finally, we investigated the interaction between 25(OH)D and BMI categories as some literature suggested [55]. Only the interaction between 25(OH)D quartiles and BMI categories was statistically significant (p = 0.021).

**Table 4:** Association between plasma 25-hydroxyvitamin D and depression symptoms (CDI ≥ 19 score) before and after adjusting for potential confounders

Vitamin D	Prevalence of depression symptoms n (%)	Crude odds ratio [95% CI]	Adjusted ^a^ odds ratio [95% CI]
25 (OH) D levels nmol/L	–	0.99 [0.98, 1.01]	1.01 [0.99, 1.02]
p-value		0.458	0.233
Q1 (25 (OH) D < 21 nmol/L) (n = 173)	27 (15.61)	[Reference]	[Reference]
Q2 (25 (OH) D ≥ 21 to < 30.35 nmol/L) (n = 179)	24 (13.41)	0.84 [0.46, 1.52]	1.07 [0.54,2.13]
Q3 (25 (OH) D ≥ 30.35 to < 45 nmol/L) (n = 175)	22 (12.27)	0.78 [0.42, 1.43]	1.27 [0.60, 2.70]
Q4 (25 (OH) D ≥ 45 nmol/L) (n = 177)	21 (11.86)	0.73 [0.39, 1.34]	1.75 [0.77, 3.99]
p-value		0.760	0.549
Severe deficiency (25 (OH) D < 25 nmol/L) (n = 250)	39 (15.60)	[Reference]	[Reference]
Deficiency (25 (OH) D ≥ 25 to < 50 nmol/L) (n = 318)	41 (12.89)	0.80 [0.50, 1.28]	1.15 [0.65, 2.06]
Insufficiency (25 (OH) D ≥ 50 to < 75 nmol/L) (n = 110)	10 (9.09)	0.54 [0.26, 1.13]	1.12 [0.47, 2.71]
Sufficiency (25 (OH) D ≥ 75 nmol/L) (n = 26)	4 (15.38)	0.98 [0.32, 3.01]	2.13 [0.59, 7.67]
p-value		0.376	0.736

Q1-Q4: quartile one to quartile four; 25 (OH) D: 25-Hydroxyvitamin D; ^a^ adjusted for all variables with p < 0.2 including gender, mother education, number of siblings, sleep hours during weekdays, sleep hours during weekend, number of times walking to from school, Body Mass Index categories, Parathyroid hormone and folate. p-value was generated by likelihood-ratio test

## Discussion

This study aimed to estimate the prevalence of depression symptoms among schoolchildren and investigate the association between 25(OH)D and depression symptoms. The prevalence of depression symptoms in schoolchildren was 13.35% and no association was found between 25(OH)D and the CDI depression score. Compared to similar studies that used the CDI and similar cut-off point score, the prevalence of depression symptoms in our setting seems to be lower than that reported from Uganda or Iran (21% and 25.6% respectively) [47, 56], higher than that reported from Cyprus (10.25%)[50], and similar to that reported from Egypt (13.3%) [57].

Our findings suggest that neither vitamin D status nor levels is associated with depression symptoms in adolescents in Middle Eastern settings. Recently, several observational studies have attempted to investigate the link between low vitamin D levels and depression or mental health problems in children and adolescents [58–71] (recently reviewed by Głąbska et al. [36]). Although the review of observational studies supported the notion that vitamin D deficiency is linked to depression or poor mental health, the studies have significant heterogeneity, which precludes meaningful summary of these studies. This included different study populations (healthy children or children with disease conditions such as renal failure, bipolar disorder, or obsessive–compulsive disorder), different methods of measurement of 25(OH)D level, and different measures of the outcome (e.g. Strengths and Difficulties Questionnaire, Beck Depression Inventory, Buss-Perry Aggression Questionnaire, Young Mania Rating Scale, Children’s Depression Rating Scale, and Mood and Feelings Questionnaire). Interventional studies that investigated the impact of vitamin D supplementation on mental health in children and adolescents [72–74] (recently reviewed by Głąbska et al. [36]) showed that vitamin D supplementation maybe beneficial for mental health. However, similar to the observational studies, there was a significant heterogeneity in these studies including study population (e.g. children with sickle cell disease or autism spectrum disorder), dosing regimens, and the outcome measurements (e.g. Depression Scale for Children, Child Behavior Checklist, Activity of Daily Living, Screen for Child Anxiety Related Emotional Disorders, Children’s Quality of Life Questionnaire, and Yale-Brown Obsessive Compulsive Scale and Children’s Depression Inventory). The review highlighted the need for more studies to facilitate comparisons and deepen the observations [36]. A recently reported randomized controlled trial (not included in the previous review) on children and adolescents, who were both vitamin D deficient and at least mildly depressed at baseline, showed that vitamin D supplementation has no effect on self-reported depression symptoms although a significant decrease of parent-reported depression symptoms was found [75].

**Fig. 2:**
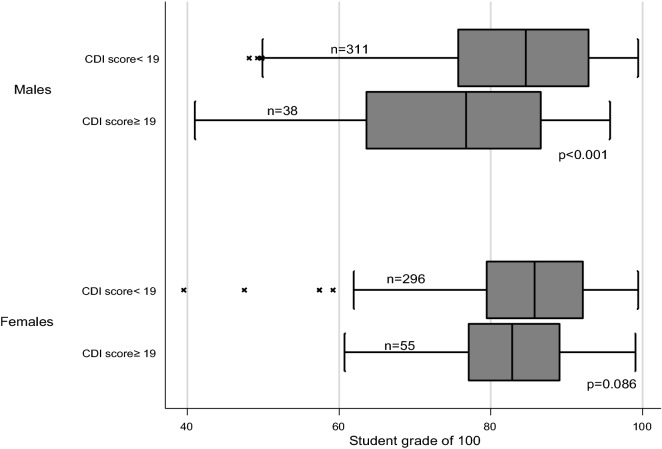
Association between depression symptoms (Children’s Depression Inventory score ≥ 19) and the students’ academic performance

In our study, the lack of association between 25(OH)D and depression symptoms may genuinely reflect the absence of the relationship between vitamin D level and depression symptoms as Mendelian randomization studies did not indicate a causal relationship between 25(OH)D concentrations and depression [76, 77]. However, it could be due to the fact that the majority (80%) of the study participants were vitamin D deficient. The other possible reason is that children with severe depression symptoms might be absent from school. This is plausible because it is postulated that vitamin D is beneficial only among those with clinically significant depression symptoms, but not in healthy participants [78] and that the presumed antidepressant effects of vitamin D may be particularly apparent at more severe stages of depression [61, 75]. Furthermore, we used the CDI, which has been validated previously in our setting. In our data, the reliability of the entire CDI scale was high (Cronbach α 0.839 [95%CI: 0.821, 0.855] and McDonald ω 0.829 [95%CI: 0.811, 0.847]. We also found that the CDI score to be related to students’ academic performance (Fig. 2), which increased our confidence in this psychometric measurement. However, it is not clear if our findings would remain the same if we used another psychometric measurement scale. It is difficult to compare our findings to the previous studies that investigated this issue using the CDI among children and adolescents. One of these studies included children with Cystic fibrosis [58] and found an association between vitamin D and depression symptoms while another study used the CDI as a measure of the outcome and found male (but not female) adolescents with major depressive disorder to have significantly lower bone mineral density as compared to healthy controls after adjusting for body mass and maturity [69]. In another study, authors used the CDI as a measure of the outcome and compared 89 depressed adolescents with 43 controls and found an association between vitamin D level and depression [79]. Finally, the lack of association between 25(OH)D and depression symptoms in our study could be attributed to different VDR genes in our setting or the complex interaction between vitamin D and both the serotonin transporter promotor gene polymorphism and childhood adversity experience [80].

There are several strengths in this study including measuring 25(OH)D using a gold standard method and collecting data from a large sample. The rigorous statistical modeling of 25(OH)D as a continuous or a categorical variable (quartiles or established cut-of points) and using quantile regression to model the CDI score as a continuous, rather than a binary outcome undoubtedly made our conclusion strong and robust. However, we did not collect data on the history of depression in the parents or the siblings, and exposure to early childhood adversity experience (such as abuse, neglect, the loss of a loved one in early life), which are potential risk factors for depression [81]. Also, like other epidemiological studies, we assessed depression symptoms using the CDI without a diagnostic interview. Finally, it is possible that many of the children with severe depression symptoms were absent from school during the study period, which may have attenuated the association.

In conclusion, we have demonstrated that one out of ten schoolchildren in Kuwait has depression symptoms and that 25(OH)D is not associated with having depression symptoms in our setting. It is important to have sufficient vitamin D levels during adolescence for several other health benefits.

## Data Availability

The data that support the findings of this study are available from the corresponding author upon reasonable request.

## Abbreviations

25(OH)D: 25-Hydroxyvitamin D
BMI: Body mass index
LC–MS/MS: Liquid chromatography-tandem mass spectrometry
PTH: Parathyroid Hormone
CDI: Children’s Depression Inventory
BDI: Beck’s Depression Inventory
